# A Unique Sandwich Structure Consisting of Graphene and Pyrene Derivatives for Ultraviolet Light Sensor

**DOI:** 10.3390/s26175405

**Published:** 2026-08-27

**Authors:** Shiyu Wang, Md. Zakir Hossain

**Affiliations:** 1 Division of Electronics and Informatics, Graduate School of Science and Engineering, Gunma University, 1-5-1 Tenjin-cho, Kiryu 376-8515, Japan; 2 Gunma University Initiative for Advanced Research (GIAR), Gunma University, 1-5-1 Tenjin-cho, Kiryu 376-8515, Japan

**Keywords:** pyrene derivative, graphene, field-effect transistor, sensor, ultraviolet light

## Abstract

We propose and demonstrate, for the first time, a sandwich structure of graphene substrates, pyrene derivatives, and an electrolyte for ultraviolet light sensing. A contact capacitor forms at the graphene–electrolyte interface via the electric double layer. When ultraviolet light excites the interlayer of the pyrene derivative, a photo-generated electric field changes the carrier density in graphene, affecting its conductivity. Thus, the sandwich structure responds to ultraviolet light intensity, which is monitored by current changes in different states. Linear fitting shows a strong positive relationship between UV intensity and current (Ids), with R^2^ above 0.99 and excellent reproducibility. The responsivity (R) and response time (*τ*_r_) for the measurement at a distance of 50 cm are estimated as 0.7 A/W and 70 s, respectively.

## 1. Introduction

Ultraviolet (UV) sensing technology has attracted significant attention in scientific research over the past few decades due to its broad applications in crime investigations, oil leak detection, fire monitoring, and power line inspections [1,2,3,4,5]. Numerous wide-band-gap semiconductor materials have been investigated for ultraviolet sensor fabrication, including III-nitrides [6], II-VI compounds [7], III-VI compounds [8], and IV-VI compounds [9]. Among these, metal oxides are of particular interest because of their abundance, excellent stability, and environmental friendliness. Additionally, metal oxide semiconductors combined with two-dimensional (2D) materials demonstrate enhanced photo-response compared to their bulk counterparts, primarily due to their large specific surface area and thinness. Recent studies have reported many promising results for UV photodetectors based on 2D materials [10].

Graphene is a two-dimensional material composed of a hexagonal lattice of sp^2^-bonded carbon atoms [11,12]. Its remarkable optical, electrical, and mechanical properties position it as a promising candidate for applications in materials science, micro- and nano-fabrication, energy, and various sensors, including graphene-based capacitors [13,14], nanopower generators [15,16], and superconductors [17,18]. Regarded by experts as a revolutionary material, graphene offers unique advantages for future technologies. In sensor development, graphene’s high carrier density and large specific surface area enable the fabrication of field-effect transistors for diverse gas and biochemical sensing applications [19,20,21,22,23,24,25,26,27,28]. Nevertheless, its atomic thinness renders it nearly transparent, allowing an absorption of only about 2.3% of incident white light [29], which poses a significant challenge for direct light detection in sensor applications. Owing to its outstanding flexibility, transparency, and ultra-thin profile, graphene holds significant promise for future development in flexible, transparent UV light sensors and photovoltaic applications.

To overcome this challenge, researchers have turned to polycyclic aromatic hydrocarbons (PAHs), a class of molecules that are typically fluorescent and emit characteristic wavelengths when exposed to light. Pyrene, a prominent PAH, is composed of four fused benzene rings, forming a flat aromatic system that exhibits blue fluorescence under ultraviolet (UV) illumination. Flat aromatic compounds and their derivatives, such as chlorine and 1-pyrenebutyric acid n-hydroxysuccinimide ester [30,31], have been widely employed to modify carbon nanotubes and graphene via surface self-assembly, a process where molecules spontaneously organize themselves on surfaces. When these flat aromatics adsorb onto graphene, fluorescence quenching reduces the fluorescence intensity [32]. This quenching typically results from Förster resonance energy transfer (FRET), a nonradiative energy transfer between molecules, and Dexter electron transfer, an energy transfer involving electron exchange. Since the typical distance between graphene and self-assembled molecules is less than 5 Å (angstroms), Dexter electron transfer predominates as the main mechanism of fluorescence quenching [33]. In this process, excitation energy is transferred from a donor to an acceptor through electron hopping. Pyrene and its derivatives are strong electron donors and can combine with materials like graphene to create electron donor–acceptor systems, which hold significant potential for applications in light sensing, energy conversion, and light harvesting.

In addition to these surface modifications, the formation of an electric double layer at the graphene–electrolyte interface is another crucial factor that influences the performance of graphene-based devices [34]. When graphene is exposed to the electrolyte, the first layer (the Stern layer) is formed by ions from the electrolyte solution that are firmly adsorbed to the graphene surface via chemical interactions. The second layer consists of ions attracted to the surface charge by Coulombic forces, electrically screening the first layer. This second layer is loosely associated with the graphene, composed of free ions that move in the fluid under the influence of electrostatic attraction and thermal motion, rather than being firmly anchored; it is thus called the “diffuse layer”. At the graphene–electrolyte interface, due to the difference in work functions, negative charge accumulates on the graphene side, while positive ions accumulate on the other side, forming the first layer of the electrical double layer [35]. The negative and positive charges are tightly bound together, and, as a result, an interfacial electric field is generated between the graphene and the electrolyte solution, forming a structure resembling an interfacial PN junction.

In this study, we introduce a pyrene derivative (1-pyrenebutyric acid n-hydroxysuccinimide ester) at the interface between graphene and the electrolyte using surface self-assembly. Upon ultraviolet (UV) irradiation, the pyrene derivative undergoes Dexter electron transfer, causing excited electrons and holes to move along the original electric field. This process generates a reverse photo-generated electric field, which weakens the existing electric field at the graphene–electrolyte interface. As a result, the conductivity of graphene is altered and can theoretically be modulated by the intensity of UV light. The primary advantage of the graphene-based UV sensor is its superior flexibility and transparency compared to conventional UV sensors. To the best of our knowledge, this is the first demonstration of a graphene UV sensor, paving the way for the development of novel transparent and flexible light sensors.

## 2. Materials and Methods

The device adopts a sandwich-like structure: the graphene substrate serves as the foundation, the pyrene derivative layer occupies the middle position, and the electrolyte liquid forms the topmost layer. Assembly proceeds from the bottom up, beginning with preparation of the graphene substrate, then modification with pyrene derivatives, and finally addition of the electrolyte liquid layer.

A 4-inch, 285 nm SiO_2_/Si wafer was purchased from ALLIANCE Biosystems (Osaka, Japan). Chemical vapor deposition (CVD) graphene foil was supplied by Chongqing Graphene Technology Co., Ltd. (Chongqing, China). Poly (methyl methacrylate) (PMMA) was purchased from Sigma-Aldrich (Tokyo, Japan), and ammonium peroxidisulfate was obtained from Kanto Chemical Co., Inc. (Tokyo, Japan). PBASE was synthesized in-house. Phosphate-buffered saline (PBS) was provided by Thermo Fisher (Waltham, MA, USA), and ultra-pure water was produced using a WL220 water purifier (Yamato, Tokyo, Japan).

3D height profiles of the interdigital electrode and substrate were measured using an OLS-4000 3D laser microscope (Olympus, Tokyo, Japan) equipped with a 405 nm laser. Raman spectroscopy was conducted on a Nicolet Almega XR spectrometer (Thermo Fisher Scientific, USA) using a 532 nm laser. X-ray photoelectron spectroscopy (xps) was performed with an AXIS-NOVA system utilizing an Al X-ray source (hv = 1486.6 eV). Real-time electrical measurements were obtained using a Keysight 4155B semiconductor parameter analyzer. The ultraviolet light source in all tests was an AS ONE LUV-16.

A 4-inch, 285 nm SiO_2_/Si wafer was diced into 1 cm × 1 cm pieces using a DISCO Corporation dicing saw (Tokyo, Japan). These SiO_2_/Si pieces served as substrates for fabricating interdigital electrodes. An interdigital shadow mask was aligned onto the SiO_2_/Si surface, and chromium/gold was chosen as the electrode material. Metal layers were deposited using electron-beam vacuum evaporation (EBVE, EIKO Engineering, Hitachinaka, Japan) to achieve thicknesses of approximately 15 nm for Cr and 50 nm for Au.

Graphene on copper foil was coated with a thin layer of PMMA using a spin coater (Mikasa Corporation, Hiroshima, Japan), then heated at 90 °C for 5 min to remove the acetone solvent. The resulting PMMA/graphene/copper foil was cut into 5 mm × 5 mm pieces suitable for the interdigital electrodes. The copper foil was etched away with an ammonium peroxidisulfate solution, leaving the PMMA/graphene film floating on the surface. After etching, the film was transferred to ultra-pure water for 30 min, then carefully placed onto the interdigitated electrode substrate. The PMMA/graphene film was air-dried at room temperature for 30 min, followed by heating at 60 °C for another 30 min. Finally, PMMA was removed with acetone and isopropyl alcohol (IPA), leaving the graphene attached to the interdigital electrodes.

To modify the graphene surface with a pyrene derivative via self-assembly, a 50 mM solution of PBASE in dry dimethylformamide (DMF) was prepared. The graphene substrate was immersed in this solution for 4 h, then washed three times with methanol and dried using a rotary pump.

A liquid reservoir was constructed on the PBASE-modified substrate using a silicone sheet, allowing for injection of the PBS solution. To form the graphene–electrolyte junction, 0.1 mL of PBS solution was injected into the reservoir and maintained at room temperature for 1 h.

All detection experiments were carried out in complete darkness to minimize ambient light interference. The ultraviolet (UV) light source was an AS ONE LUV-16. During measurements, the drain voltage was set to 1 V, with both the source and back gate terminals grounded. Real-time current was monitored using a Keysight 4155B semiconductor parameter analyzer. Measurements were performed three times at distances of 50 cm, 70 cm, and 100 cm between the UV light source and the device, enabling the analysis of device response under different light intensities. Each test lasted up to 1000 s. The UV light source was switched on during intervals of 100–200 s, 400–500 s, and 700–800 s; it remained off at all other times.

## 3. Results and Discussion

**Figure 1:**
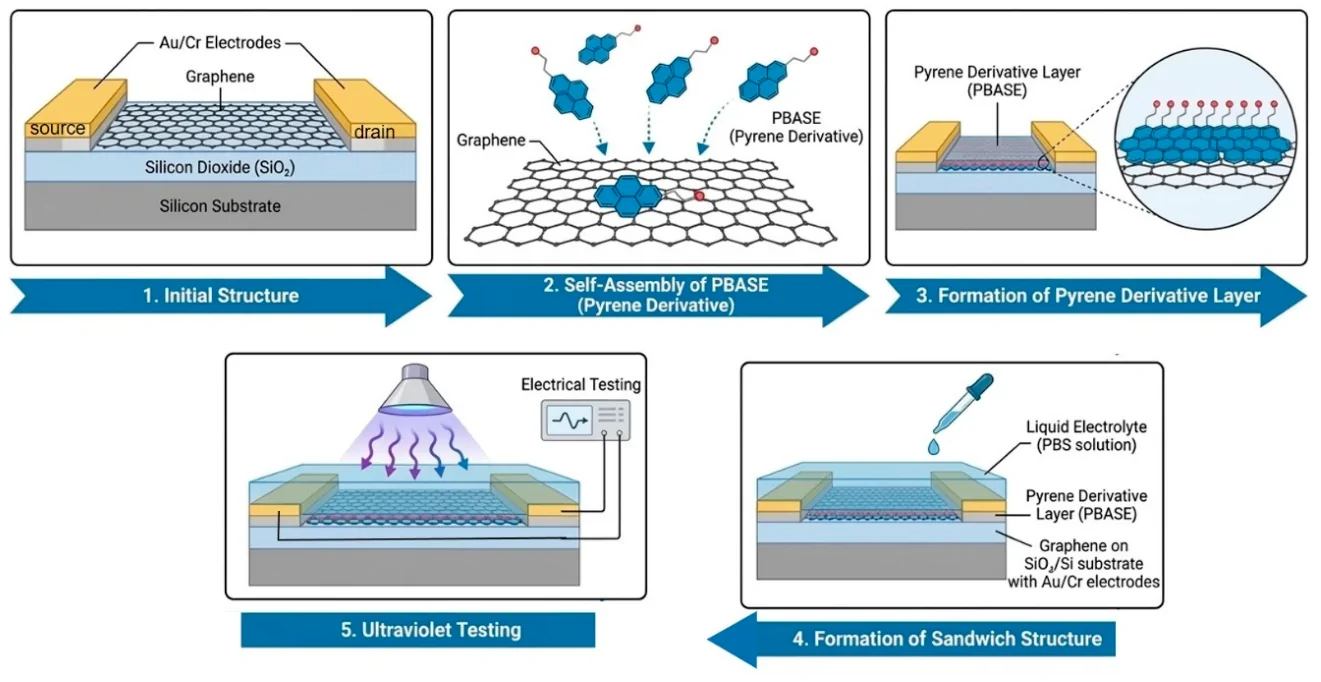
Schematic of fabrication and testing workflow of a graphene-based device covered with PBASE and evaluated under UV light.

The device consists of three primary layers: a graphene layer, a pyrene derivative layer, and a liquid electrolyte layer. The details of the fabrication and characterization of the device under UV light irradiation are illustrated in Figure 1. Pyrene derivatives were self-assembled onto the graphene and were expected to form a uniform layer, as depicted in the panel [36]. The π electron cloud of the pyrene group interacted with the π cloud of graphene, resulting in the π−π overlap forming a uniform self-assembled monolayer onto the graphene [36]. Following the addition of the electrolyte (PBS), the device was characterized through two-terminal measurements under UV light ON and OFF states.

**Figure 2:**
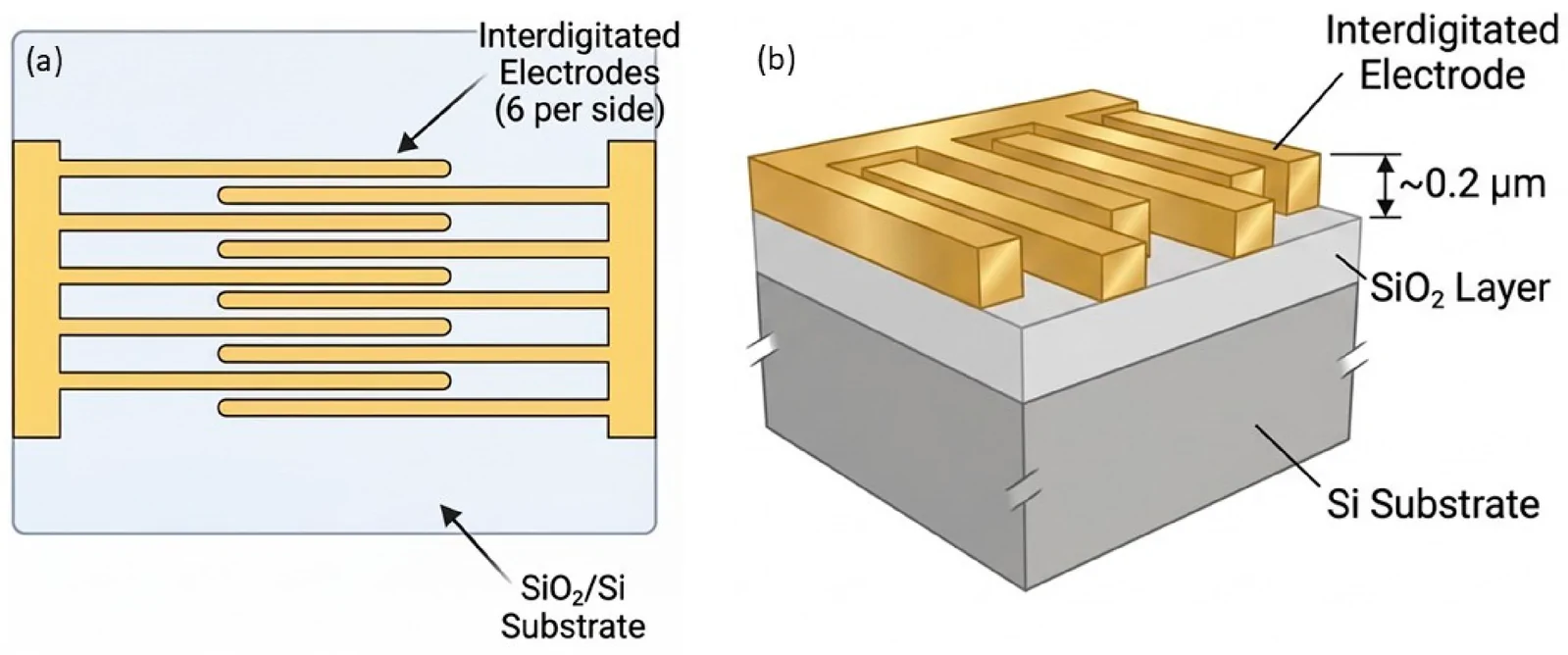
Schematic of interdigitated electrodes assembly: (**a**) top-down view and (**b**) a cross-section.

Figure 2a presents optical images of the interdigiated electrodes assembly fabricated on a SiO_2_/Si substrate (a silicon wafer with a thin layer of silicon dioxide). Six pairs of these electrodes were created on a 1 cm × 1 cm substrate, each approximately 200 μm wide with 200 μm gaps between them. According to the 3D height profile, the thickness of the Cr/Au (chromium/gold) electrode layer is about 20 nm.

**Figure 3:**
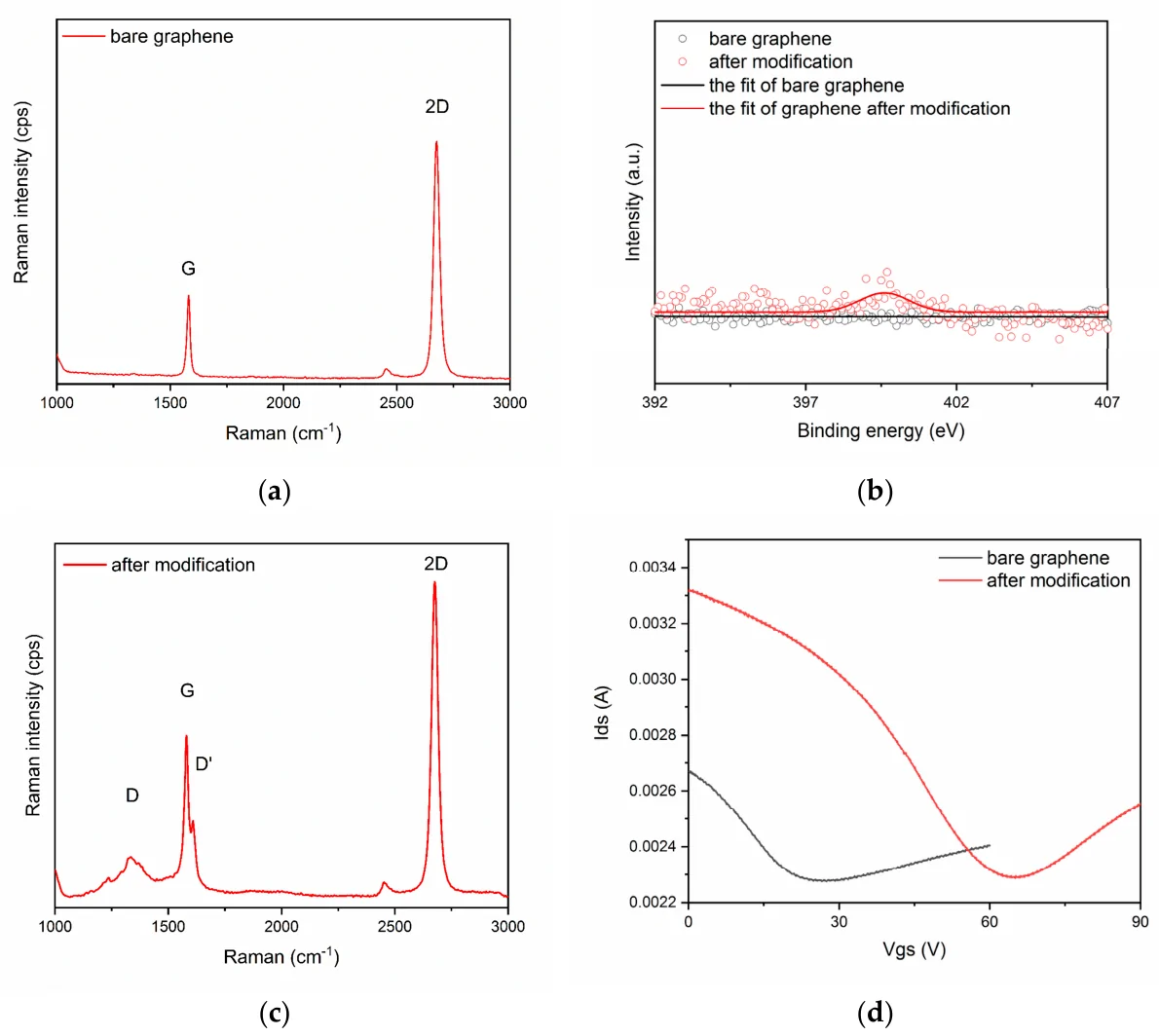
(**a**) The Raman spectroscopy of the bare graphene substrate. (**b**) The narrow region xps spectra covering the nitrogen (N 1s) peak before and after bare graphene substrate modification. (**c**) Raman spectroscopy of the graphene substrate after modification. (**d**) The I-V characteristics of the bare graphene and after modification.

To verify the quality of the transferred graphene, Raman measurements were performed at random locations. Figure 3a displays a typical Raman spectrum, where the two main peaks, 2D (near 2700 cm^−1^) and G (around 1580 cm^−1^), demonstrate features of high-quality graphene. The D band, near 1300 cm^−1^, signifies lattice defects, and its low intensity indicates a clean sample. The intensity ratio I(2D)/I(G) exceeds 2, consistent with monolayer graphene [37].

1-pyrenebutyric acid N-hydroxysuccinimide ester (PBASE) is an effective pyrene derivative frequently used as a chemical linker for attaching graphene and carbon nanotubes. As a linker, it forms stable bonds between materials. When attached to graphene, it introduces nitrogen atoms, which are absent in pristine graphene and can be detected using X-ray photoelectron spectroscopy (xps). To confirm successful modification, we utilized xps for surface elemental analysis, current–voltage (IV) characterization for electrical properties, and Raman spectroscopy for vibrational structure.

Figure 3b presents the N 1s X-ray photoelectron spectroscopy (xps) spectrum of graphene after modification with 1-pyrenebutyric acid N-hydroxysuccinimide (NHS) ester. A weak N 1s peak appears near 400 eV after modification, whereas no such peak is observed before. This confirms that the pyrene derivative binds to the graphene surface through self-assembly.

Figure 3c shows the Raman spectrum of graphene after modification, revealing the appearance of D (1300 cm^−1^) and D′ (1620 cm^−1^) bands compared to bare graphene. These bands correspond to vibrational modes that signal increased structural disorder in the sp^2^-hybridized carbon system, a phenomenon reported in previous studies [38,39].

Current–voltage (IV) measurements before and after modification (Figure 3d) show that the Dirac point shifts to higher energies upon the addition of PBASE, indicating effective p-type doping of the graphene. Helmholtz invented the first model of the electric double layer. An electric double layer forms at the interface between an electrode and an electrolyte, where charges arrange themselves into two distinct layers. He modeled this system as a simple capacitor, meaning two layers of charge separated by a small distance. Later, Louis Georges Gouy and David Chapman introduced the diffuse model, in which the electric potential (the energy required to move a charge in an electric field) decreases exponentially with distance from the surface. However, the Gouy–Chapman model does not work for highly charged double layers. Stern later improved this model by combining the Helmholtz and Gouy–Chapman models, resulting in an internal Stern layer (similar to Helmholtz’s capacitor, representing immobile ions near the surface) and an outer diffuse layer (the Gouy–Chapman layer, with mobile ions). Most researchers now use this combined model.

At the interface between graphene and the electrolyte solution, an electric double layer forms, consisting of two distinct charge layers (Figure 1). Simulations and experiments indicate that the graphene side attracts electrons, while the electrolyte side attracts cations [15,35]. The first layer, known as the Stern layer, consists of tightly coupled electronic and cationic layers, forming a capacitor-like boundary that stores charge. This generates an intrinsic electric field at the interface. Graphene–electrolyte interfaces have been used to develop nanogenerators, which convert mechanical or chemical energy into electricity [5,25]. The voltage generated at this interface can be regulated, and the structure resembles a PN junction at the solid–liquid boundary.

The Dexter exchange mechanism requires direct contact between donor and acceptor molecules, with energy transfer occurring efficiently when their separation is less than 5 Å. In our system, the typical distance between graphene and polycyclic aromatic hydrocarbons is about 3 Å [38]. Recent research confirms that Dexter exchange is the dominant nonradiative energy transfer pathway between graphene and the PBASE complex, surpassing Förster resonance energy transfer [33].

In present experiment, when the pyrene derivative layer is exposed to ultraviolet radiation, electron–hole pairs are generated via the Dexter exchange mechanism within the capacitor-like structure. These carriers move along the intrinsic electric field, leading to recombination of the original electrons and cations at the interface and reducing the total charge. The photo-generated field thus opposes and weakens the original electric field at the interface.

**Figure 4:**
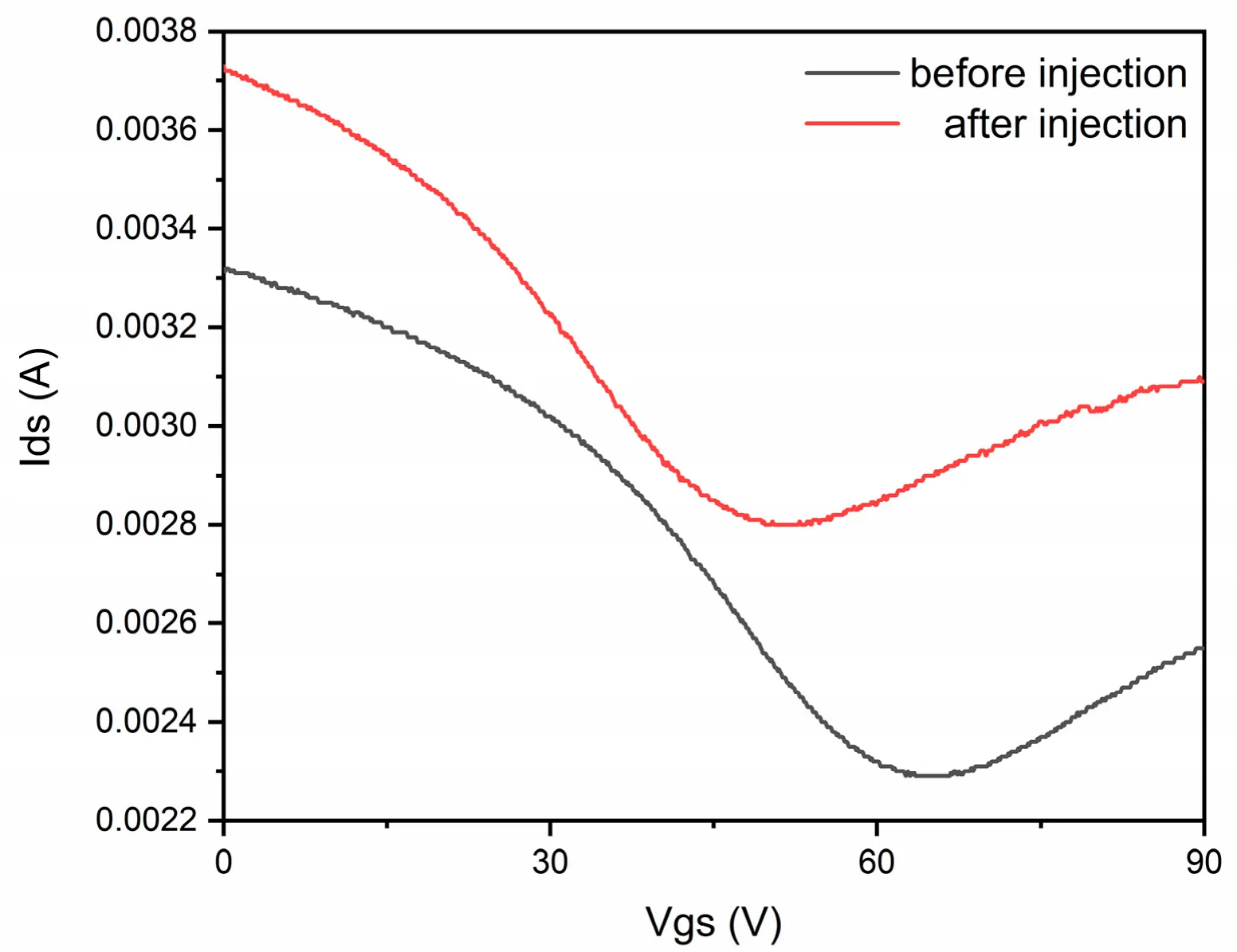
I-V curves of pyrene modified graphene before and after injection of electrolyte solution.

Figure 4 shows that, after injecting the electrolyte solution, the Dirac point (the gate voltage at which graphene’s charge carriers switch from electrons to holes) shifts in the positive direction, moving slightly to the left of its pre-injection position. We can therefore confirm that graphene is p-type (having holes as the majority charge carriers) when the original interface electric field forms, and the hole concentration is slightly reduced due to electron accumulation on the graphene side. Since graphene remains p-type after injection of the electrolyte solution, holes are the dominant carriers. Theoretically, when ultraviolet light reduces electronic doping in graphene (decreases the number of electrons added to graphene) and we ground the back gate, hole conductivity will increase and, accordingly, the Ids current (drain–source current) will increase.

**Figure 5:**
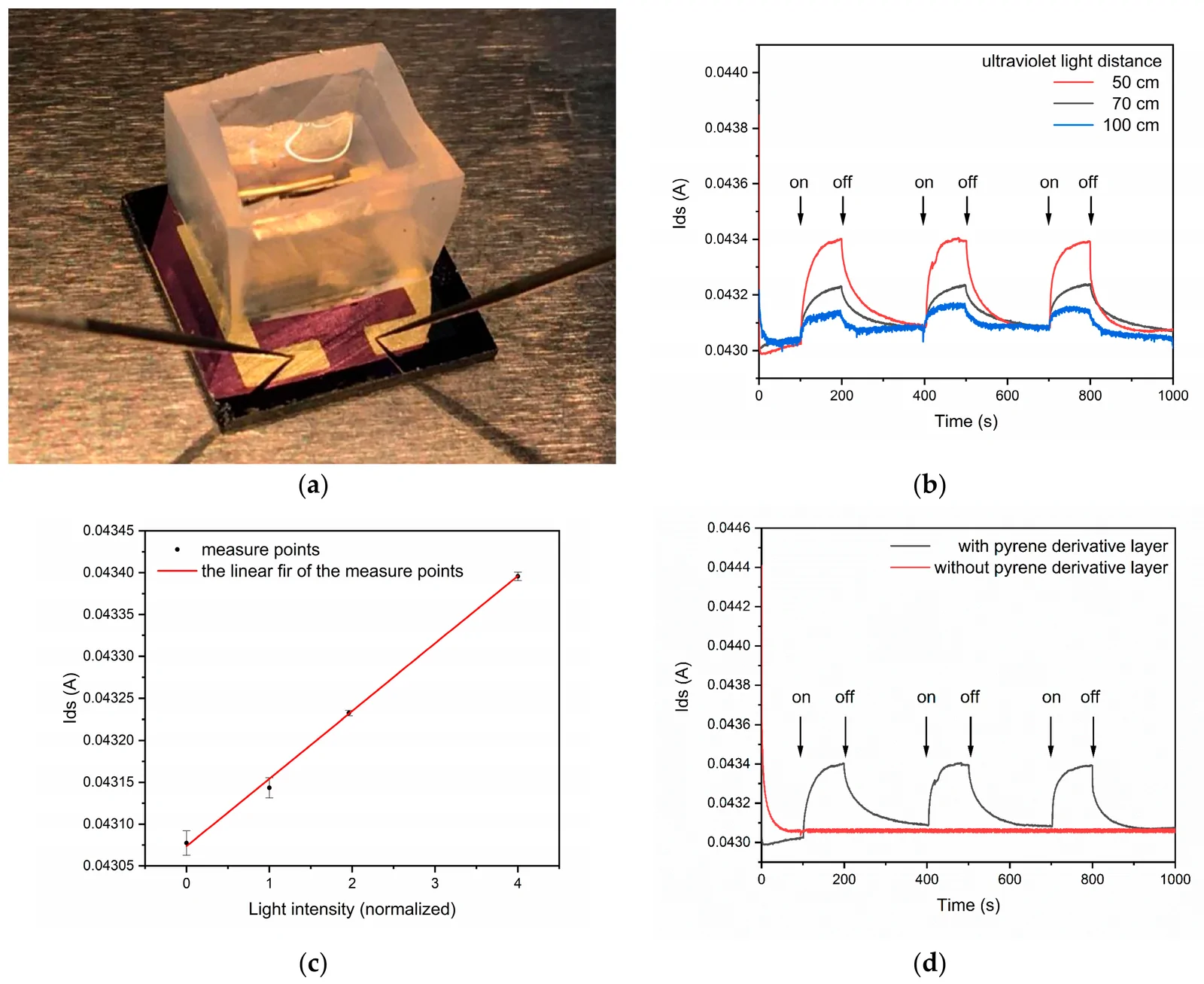
(**a**) Optical image of a device used for the real-time current (Ids) measurement upon irradiation of uv light. (**b**) Real-time Ids values upon uv source on and off states. (**c**) Linear fitting of the change in Ids value with uv intensity. (**d**) Real-time Ids values with and without the pyrene derivative layer (control experiment).

Figure 5a shows a schematic of the device used in the present study for characterizing the ultraviolet sensitivity by measuring real-time current through two terminals. For holding the liquid electrolyte, the silicone pool was constructed upon the electrodes.

Figure 5b presents real-time measurements of the sensor’s response to varying UV light intensities. Activation of the UV source produces a marked increase in current between the source and drain electrodes, which returns to baseline when the light is switched off. Repeated on/off cycles demonstrate excellent reproducibility, with the sensor delivering consistent current responses for each cycle. These results confirm the sensor’s reliable and repeatable response to UV radiation.

To analyze the relationship between ultraviolet (UV) light intensity and the sensor’s output current (Ids), we selected data points after signal stabilization. UV light intensity was varied by adjusting the distance between the light source and the sensor, with intensities corresponding to X, 1.96X, and 4X for distances of 100 cm, 70 cm, and 50 cm, respectively. Figure 5c shows that, after normalization, Ids (Y) and light intensity (X) maintain a linear relationship, described by Y = 8.06154E-5*X + 0.04307, with a coefficient of determination (R^2^) above 0.99. This confirms that Ids increases linearly with UV light intensity.

The increase in the Ids value upon irradiation with UV light is normally termed as photocurrent (I_ph_). The photocurrent of a material depends on the power (i.e., intensity) of the UV irradiation. The I_ph_ of a photodetector device can be expressed by I_ph_ = I_uv_ − I_dark_, where I_uv_ is the current under UV irradiation, and I_dark_ is the current under a dark environment. The irradiation output of the UV light source used in this experiment is 4000 mW/cm^2^. The photocurrent I_ph_ during the irradiation at the distance of 50 cm is estimated to be 0.3 mA. Note that I_ph_ depends on the intrinsic properties of channel materials, power of irradiation, and the geometry of the device. The effective photo-irradiated area of the current device is estimated to be 0.3 cm^2^. For the characterization of the current device, the photo-responsivity (R) and response times are also estimated. Photo-responsivity can be expressed by R = I_ph_/(P_in_ * S), where P_in_ is the UV power per unit area (mW/cm^2^) and S is the effective area of the device. The response time includes two terms, the rising time (*τ*_r_) and falling time (*τ*_f_), which are defined as the time required for increasing the Ids values from 10% to 90% and decreasing the Ids values from 90% to 10% of the photocurrent, respectively. The responsivity (R) and response time (*τ*_r_) for the measurement at the distance of 50 cm are estimated to be 0.7 A/W and 70 s, respectively. The response times for reduced graphene oxides estimated from three terminal measurements were reported as 2 s and 100 s, while the responsivity was reported to be 4 mA/W and 0.12 A/W [39,40]. Note that the response (*τ*_r_) and falling (*τ*_f_) times of a photodetector depend on many factors, including the intrinsic properties of the photoconductor, light wavelength, nature and concentration of defects, temperature, electrode geometry, and humidity [41].

As a control, we tested a graphene ultraviolet sensor without the pyrene derivative layer under identical conditions (UV source at 50 cm). Figure 5d demonstrates that the current (Ids) remains constant regardless of UV illumination. Similar non-responsive Ids values were also observed for the graphene/electrolyte system. These confirm that the observed current changes in the main device are attributable to the pyrene derivative layer rather than the graphene–electrolyte junction or other factors. The device is found to be robust in ambient conditions. Even after a week of making the device, a similar UV response was observed.

Significant advances in the design and fabrication of photoelectric UV sensors have been achieved over the past decade. With a range of platforms now available for UV detection, here we discuss a concise overview of key technologies in photoelectric UV sensors. Currently, zinc oxide (ZnO) and carbon nanotubes (CNTs) are among the most extensively studied materials for UV sensor development, especially in the context of wearable technologies. ZnO-based materials, such as nanowires [42], nanorods [43], and nanospheres [44], stand out for their straightforward fabrication and tunable morphologies, which enable precise control over optical properties. As a result, ZnO has emerged as a leading building block for UV sensors, with nanowires (NWs) garnering particular interest due to their versatile morphologies. Similarly, carbon-based materials like CNTs offer a unique blend of electrical and optical properties, making them highly attractive for sensing applications, especially in environmental monitoring [45]. Their high surface area, hollow structure, and distinctive chemical and electrical characteristics further enhance their suitability for UV sensor applications [46]. Recently, our group has demonstrated graphene oxide-derived UV detection with a fast response time and high quantum efficiency [47].

Graphene, as another carbon-based material, shares many attributes with CNTs, such as an atomically smooth surface and unique chemical and electrical properties [11]. Unlike ZnO, graphene can be fabricated into various patterns with greater ease and long-term stability using established semiconductor processing methods [42]. These observations strongly suggest that novel and advanced UV light sensors based on graphene are poised for significant development in the near future.

## 4. Conclusions

We have shown that a graphene-based sensor modified with a pyrene derivative, arranged in a sandwich structure with an electrolyte, effectively detects ultraviolet light. The sensor exhibits excellent repeatability and ambient stability. The irradiation of ultraviolet light onto the sensor was detected by the enhancement of Ids (current passes through the graphene channel), which follows a clear linear relationship with UV intensity. To our knowledge, this is the first successful application of such a sandwich-structure sensor (graphene, pyrene derivative, electrolyte) for ultraviolet sensing. The quantitative analysis of Ids values upon irradiation of UV light results in responsivity (R) and response time (*τ*_r_) values for a typical measurement at a distance of 50 cm of 0.7 A/W and 70 s, respectively.

## Data Availability

The data presented in this study are available on request from the corresponding author due to privacy.
